# Subchronic oral toxicity of silver nanoparticles

**DOI:** 10.1186/1743-8977-7-20

**Published:** 2010-08-06

**Authors:** Yong Soon Kim, Moon Yong Song, Jung Duck Park, Kyung Seuk Song, Hyeon Ryol Ryu, Yong Hyun Chung, Hee Kyung Chang, Ji Hyun Lee, Kyung Hui Oh, Bruce J Kelman, In Koo Hwang, Il Je Yu

**Affiliations:** 1 Korea Environment & Merchandise Testing Institute, Incheon, Korea; 2 College of Medicine, Chung-Ang University, Seoul, Korea; 3 Chemcial Safety and Health Research Center, KOSHA, Daejeon, Korea; 4 College of Medicine, Kosin University, Busan, Korea; 5 Korea Agency for Technology and Standards, Gwacheon, Korea; 6 Veritox, Inc., Seattle, USA; 7 College of Veterinary Medicine, Seoul National University, Seoul, Korea; 8 Fusion Technology Research Institute, Hoseo University, Asan, Korea

## Abstract

**Background:**

The antibacterial effect of silver nanoparticles has resulted in their extensive application in health, electronic, consumer, medicinal, pesticide, and home products; however, silver nanoparticles remain a controversial area of research with respect to their toxicity in biological and ecological systems.

**Results:**

This study tested the oral toxicity of silver nanoparticles (56 nm) over a period of 13 weeks (90 days) in F344 rats following Organization for Economic Cooperation and Development (OECD) test guideline 408 and Good Laboratory Practices (GLP). Five-week-old rats, weighing about 99 g for the males and 92 g for the females, were divided into four 4 groups (10 rats in each group): vehicle control, low-dose (30 mg/kg), middle-dose (125 mg/kg), and high-dose (500 mg/kg). After 90 days of exposure, clinical chemistry, hematology, histopathology, and silver distribution were studied. There was a significant decrease (P < 0.05) in the body weight of male rats after 4 weeks of exposure, although there were no significant changes in food or water consumption during the study period. Significant dose-dependent changes were found in alkaline phosphatase and cholesterol for the male and female rats, indicating that exposure to more than 125 mg/kg of silver nanoparticles may result in slight liver damage. Histopathologic examination revealed a higher incidence of bile-duct hyperplasia, with or without necrosis, fibrosis, and/or pigmentation, in treated animals. There was also a dose-dependent accumulation of silver in all tissues examined. A gender-related difference in the accumulation of silver was noted in the kidneys, with a twofold increase in female kidneys compared to male kidneys.

**Conclusions:**

The target organ for the silver nanoparticles was found to be the liver in both the male and female rats. A NOAEL (no observable adverse effect level) of 30 mg/kg and LOAEL (lowest observable adverse effect level) of 125 mg/kg are suggested from the present study.

## Background

The antibacterial activity exhibited by silver in a range of studies [1-5] has resulted in the widespread use of silver nanoparticles in bedding, washing machines, water purification, toothpaste, shampoo and rinse, nipples and nursing bottles, fabrics, deodorants, filters, kitchen utensils, toys, and humidifiers [6,7], where the main body or inner surface of the product is mixed or coated with germ-resistant nano-silver to prevent the growth of fungi and bacteria. Despite such widespread use of silver-nanoparticle-containing products, subchronic and chronic toxicity data on silver nanoparticles remain rare. The lack of exposure data on silver nanoparticles in the workplace and silver nanoparticles released from consumer products or released into the environment makes it difficult to assess the risks of using these materials. Limited data have been reported for silver nanoparticles by inhalation and oral routes of exposure. The target organs for silver nanoparticles by subchronic inhalation are the lungs and liver in male and female rats [8]. This study suggested a NOAEL (no observable adverse effect level) of 100 μg/m^3^.

A LOAEL (lowest observable adverse effect level) and NOAEL have been reported for a 28-day oral toxicity study using Sprague-Dawley rats to be 300 mg/kg and 30 mg/kg, respectively [9]. In this study, F344 rats were exposed to silver nanoparticles following the Organization for Economic Cooperation and Development (OECD) test guideline 408 (OECD, 1998), using a 13-week repeated-oral-dose toxicity protocol. The study was conducted under OECD Good Laboratory Practices (GLP). Clinical chemistry, histopathology, and distribution of silver nanoparticles were investigated in blood, lungs, kidneys, brain, liver, and other organs.

## Materials and methods

### Silver Nanoparticles

**Figure 1:**
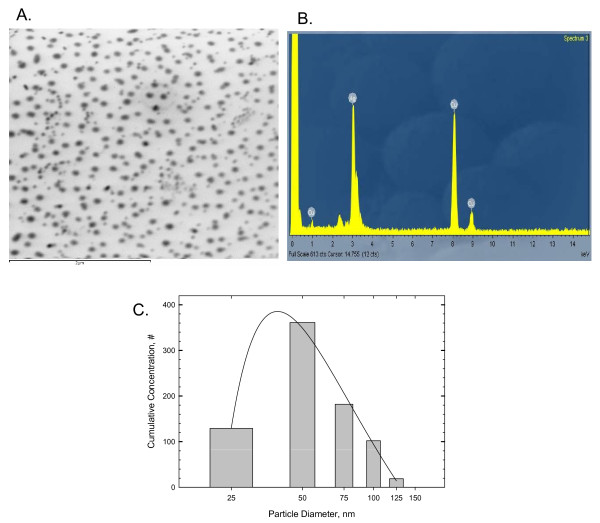
**Transmission electron micrograph of silver nanoparticles and distribution of silver nanoparticles**. The bar indicates 2 μm. A. Transmission electron micrograph of silver nanoparticles. B. Energy dispersive x-ray profile (silver nanoparticles on copper grid). C. Distribution of silver nanoparticles.

Silver nanoparticles (CAS No. 7440-22-4) were purchased from NAMATECH, Ltd. (Daejeon, Korea), and were at least 99.98% pure. Count median diameter and geometric standard deviation of silver nanoparticles in 0.5% aqueous carboxymethylcellulose (CMC, Sigma USA) analyzed by transmission electron microscopy were 56 nm and 1.46, respectively (Figure 1).

### Transmission Electron Microscopy

The filters on which the silver nanoparticles in the 0.5% CMC were filtered were coated with carbon, mounted on an electron microscope grid (200 mesh, Veco, Eerbeek, Holland), and visualized under a transmission electron microscope (TEM, Hitachi 7100). The diameters of 400 randomly selected particles were measured at 50,000 × magnification, and the silver particles were analyzed using an energy-dispersive x-ray analyzer (EDX-200, Horiba, Japan) at an accelerating voltage of 75 kV.

### Animals and Conditions

Four-week-old male and female, specific-pathogen free (SPF) Fisher 344 rats were purchased from Japan SLC Inc. (Japan) and acclimated for 7 days before starting the experiments. During the acclimation and experimental periods, the rats were housed in polycarbonate cages (maximum of 3 rats per cage) in a room with controlled temperature (22.2 ± 1.7°C) and humidity (48.4 ± 6.0%), and a 12-h light/dark cycle. The rats were fed a rodent diet (Harlan Teklad, USA) and filtered water *ad libitum*. The rats were divided into 4 groups (10 rats in each group): vehicle control (0.5% carboxymethylcellulose, CMC), low-dose group (30 mg/kg/day), middle-dose group (125 mg/kg/day), and high-dose group (500 mg/kg/day). When the rats reached five weeks of age, they were exposed to silver nanoparticles following OECD test guideline 408 [10] by gavage for 13 weeks of repeated oral administration (dosing volumes were 10 ml/kg). Dose levels were selected based on previous observations in a 28-day oral toxicity study by Kim et al. [9]. The study was conducted under OECD Good Laboratory Practices.

### Clinical Chemistry and Hematology

At the conclusion of the 13-wk experiment, the rats were 18 wks old. Before necropsy, food was withheld for 24 h and the rats were anesthetized with CO_2 _gas. Blood was then drawn from the abdominal aorta, collected in heparinized vacutainers, and analyzed for ALB (albumin), ALP (alkaline phosphatase), Ca (calcium), CHO (cholesterol), CRE (creatinine), gamma-GT (gamma-glutamyl transpeptidase), GLU (glucose), GOT (glutamic oxaloacetic transaminase), GPT (glutamic pyruvic transminase), IP (inorganic phosphorus), LDH (lactate dehydrogenase), MG (magnesium), TP (total protein), UA (uric acid), BUN (blood urea nitrogen), TBIL (total bilirubin), CK (creatine phosphokinase), Na (sodium), K (potassium), Cl (chloride), TG (triglyceride), and A/G (ratio of albumin to globulin) using a biochemical blood analyzer (Hitachi 7180, Hitachi, Japan). The blood was also analyzed for the WBC (white blood cell count), RBC (red blood cell count), Hb (hemoglobin concentration), HTC (hematocrits), MCV (mean corpuscular volume), MCH (mean corpuscular hemoglobin), MCHC (mean corpuscular hemoglobin concentration), RDW (red cell distribution width), PLT (platelet count), MPV (mean platelet volume), NE# (number of neutrophils), NE% (percent of neutrophils), LY# (number of lymphocytes), LY% percent of lymphocytes), MO# (number of monocytes), MO% (percent of monocytes), EO# (number of eosinophils), EO% (percent of eosinophils), BA# (number of basophils), and BA% (percent of basophils) using a blood cell counter (Hemavet 0950, CDC Tech., USA).

### Organ Weights and Histopathology

After collecting blood samples, the rats were killed by cervical dislocation. Adrenal glands, bladder, testes, ovaries, uterus, epididymis, seminal vesicle, heart, thymus, thyroid gland, trachea, esophagus, tongue, prostate, lungs, nasal cavity, kidneys, spleen, liver, pancreas, and brain were removed carefully, weighed, and fixed in a 10% formalin solution containing neutral phosphate-buffered saline. Thereafter, the organs were embedded in paraffin, stained with hematoxylin and eosin, and examined under light microscopy.

### Determination of Tissue Silver

Tissues were digested with conc. nitric acid by using a microwave digestion system (MARS 230/60, CEM). The concentration of silver in digested fluid was analyzed with a flameless method using an atomic absorption spectrophotometer equipped with a Zeeman graphite furnace (Perkin Elmer 5100ZL, Zeeman Furnace Module, USA) based on the NIOSH 7300 method [11]. The concentration of silver in the tissue was expressed as μg/g wet weight.

### Statistical Analysis

Statistical analysis was performed with SPSS (Version 12). Statistical evaluation was performed by analysis of two-tailed Student's t-test or analysis of variance (ANOVA) following multiple comparison tests with Duncan's method. The level of statistical significance was set at p < 0.05

## Results

### Animal Observation, Food Consumption, and Effect on Body and Organ Weights

**Figure 2:**
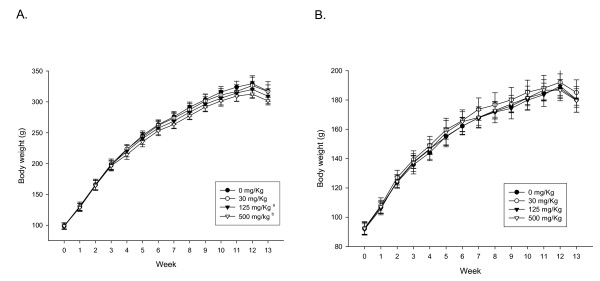
**Body weight changes during 90-day oral administration of silver nanoparticles**. A. Male. a Significant difference vs. control, p < 0.05. (10 weeks). b Significant difference vs. control, p < 0.05. (4, 5, 7~13 weeks). B. Female.

There were no significant differences in food consumption and water intake between treated male and female rats and the control group (data not shown). There were no significant dose-related changes in the body weight of female rats; however, there were significant (P < 0.05) dose-related decreases in the body weight of high-dose male rats at 4, 5, and 7 weeks of exposure at the conclusion of the study 13 weeks, and middle-dose male rats at 10 weeks of exposure (Figure 2). No significant organ-weight changes were observed in either the male or female rats after 90 days except for an increase (P < 0.05) in the weight of the left testis for the high-dose male rats, and for decreases (P < 0.05) in the weight of right kidney for the low-and middle-dose female rats (Tables 1 and 2).

**Table 1:** Relative organ weights for male rats after 90-day oral administration of silver nanoparticles (mean ± S.D., n≥9)

	Dose (mg/Kg)			
	
	0	30	125	500
Testis (Left)	0.47 ± 0.02	0.48 ± 0.03	0.49 ± 0.02	0.51 ± 0.01*

Testis (Right)	0.47 ± 0.02	0.48 ± 0.02	0.48 ± 0.02	0.49 ± 0.02

Spleen	0.21 ± 0.03	0.21 ± 0.01	0.22 ± 0.01	0.20 ± 0.02

Liver	2.96 ± 0.10	2.91 ± 0.16	2.92 ± 0.09	2.87 ± 0.11

Pituitary gland	0.002 ± 0.001	0.003 ± 0.001	0.005 ± 0.006	0.004 ± 0.003

Adrenal gland (Left)	0.011 ± 0.006	0.007 ± 0.002	0.008 ± 0.001	0.009 ± 0.003

Adrenal gland (Right)	0.008 ± 0.001	0.007 ± 0.001	0.008 ± 0.001	0.009 ± 0.002

Prostate	0.13 ± 0.06	0.16 ± 0.05	0.15 ± 0.04	0.16 ± 0.05

Lungs	0.42 ± 0.02	0.41 ± 0.04	0.42 ± 0.03	0.43 ± 0.03

Brain	0.62 ± 0.02	0.61 ± 0.03	0.62 ± 0.02	0.63 ± 0.02

Heart	0.27 ± 0.02	0.26 ± 0.02	0.27 ± 0.01	0.26 ± 0.02

Thymus	0.10 ± 0.02	0.10 ± 0.02	0.11 ± 0.02	0.10 ± 0.02

Kidney (Left)	0.28 ± 0.01	0.28 ± 0.02	0.29 ± 0.01	0.28 ± 0.01

Kidney (Right)	0.29 ± 0.02	0.28 ± 0.02	0.29 ± 0.01	0.29 ± 0.01

*Significant difference vs. control, *p *< 0.05

**Table 2:** Relative organ weights for female rats after 90-day oral administration of silver nanoparticles (mean ± S.D., n≥9)

	Dose (mg/Kg)			
	
	0	30	125	500
Ovary (Left)	0.027 ± 0.004	0.026 ± 0.005	0.023 ± 0.003	0.024 ± 0.007

Ovary (Right)	0.072 ± 0.134	0.025 ± 0.006	0.026 ± 0.003	0.028 ± 0.005

Spleen	0.22 ± 0.02	0.21 ± 0.01	0.21 ± 0.01	0.21 ± 0.02

Liver	2.67 ± 0.18	2.61 ± 0.07	2.53 ± 0.12	2.62 ± 0.17

Pituitary gland	0.007 ± 0.002	0.006 ± 0.002	0.006 ± 0.001	0.007 ± 0.002

Adrenal gland (Left)	0.015 ± 0.003	0.014 ± 0.002	0.014 ± 0.001	0.015 ± 0.003

Adrenal gland (Right)	0.014 ± 0.001	0.014 ± 0.002	0.014 ± 0.002	0.015 ± 0.001

Uterus	0.233 ± 0.044	0.257 ± 0.090	0.216 ± 0.040	0.195 ± 0.040

Lungs	0.54 ± 0.06	0.55 ± 0.04	0.50 ± 0.05	0.54 ± 0.05

Brain	0.99 ± 0.05	0.98 ± 0.05	0.99 ± 0.06	1.00 ± 0.05

Heart	0.32 ± 0.02	0.32 ± 0.02	0.31 ± 0.02	0.32 ± 0.03

Thymus	0.14 ± 0.02	0.13 ± 0.02	0.13 ± 0.02	0.15 ± 0.02

Kidney (Left)	0.31 ± 0.02	0.30 ± 0.02	0.30 ± 0.01	0.30 ± 0.02

Kidney (Right)	0.31 ± 0.02	0.29 ± 0.01*	0.30 ± 0.01*	0.30 ± 0.02

*Significant difference vs. control, *p *< 0.05

### Effects on Clinical Chemistry and Hematology

**Figure 3:**
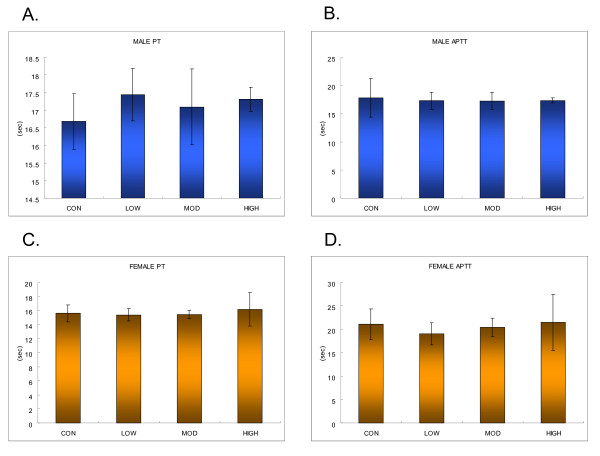
**Coagulation times for male and female rats: A, male prothrombin time (PT); B, male active partial thromboplastine time (APPT); C, female PT; D, female APPT**. (Error bars indicate standard deviation.)

There appeared to be increase ALP for male rats in the middle and high-dose groups statistically not significant (Table 3). However, there was a significant increase (P < 0.01) in alkaline phosphatase (ALP) for female rats in the high-dose groups (Table 4). A significant increase (P < 0.01) in cholesterol was also found in the middle and high-dose male rats and the high-dose female rats (Table 3 and 4). A significant increase (P < 0.05) in total bilirubin was noted in the middle-dose male rats (Table 3). A significant decrease in magnesium, protein, and inorganic phosphorus was found in the middle and high-dose female rats (Table 4). No significant changes in the hematological parameters were noted except for a significant increase (P < 0.05) in monocytes in the high-dose female rats (Table 5 and 6). The reticulocyte count for the low-dose female rats decreased significantly (p < 0.05) when compared with the control group (Table 7). Coagulation time in terms of the active partial thromboplastin time (APTT) and prothrombin time (PT) did not show any significant changes when compared with the control group (Figure 3).

**Table 3:** Serum values for male rats after 90-day oral administration of silver nanoparticles (mean ± S.D.)

	Dose (mg/Kg)			
	
	0 (n = 10)	30 (n = 10)	125 (n = 10)	500 (n = 10)
ALB	2.92 ± 0.26	2.82 ± 0.13	2.79 ± 0.10	2.86 ± 0.10

ALP	291.60 ± 35.40	279.50 ± 48.78	306.40 ± 35.16	343.80 ± 65.67

CA	10.93 ± 0.59	10.77 ± 0.67	10.49 ± 0.22	10.60 ± 0.24

CHO	88.50 ± 8.45	94.80 ± 7.54	98.30 ± 5.46**	106.00 ± 6.38**

CRE	0.91 ± 0.10	0.86 ± 0.11	0.85 ± 0.05	0.83 ± 0.15

GGT	0.40 ± 0.52	0.10 ± 0.32	0.40 ± 0.52	0.50 ± 0.53

GLU	168.20 ± 24.36	193.50 ± 42.15	165.40 ± 17.66	177.70 ± 20.89

AST	105.00 ± 27.01	97.10 ± 22.71	85.00 ± 12.35	98.20 ± 35.64

ALT	72.10 ± 5.17	77.20 ± 8.39	68.70 ± 4.64	71.40 ± 20.03

LDH	1230.80 ± 1351.96	784.80 ± 675.03	598.70 ± 351.37	644.20 ± 400.41

MG	2.27 ± 0.33	2.36 ± 0.62	2.06 ± 0.10	2.19 ± 0.09

TP	6.61 ± 0.21	6.51 ± 0.23	6.42 ± 0.19	6.56 ± 0.21

UA	1.19 ± 0.73	1.33 ± 1.21	0.79 ± 0.29	0.76 ± 0.22

BUN	22.14 ± 1.68	21.60 ± 2.59	22.47 ± 1.89	22.76 ± 2.93

T-BIL	0.009 ± 0.013	0.006 ± 0.008	0.024 ± 0.017*	0.013 ± 0.014

IP	6.99 ± 0.82	6.98 ± 1.06	6.40 ± 0.51	6.59 ± 0.56

TG	140.20 ± 42.35	156.30 ± 49.69	161.40 ± 44.60	140.40 ± 47.28

CPK	554.10 ± 598.27	342.60 ± 267.05	269.10 ± 128.28	273.50 ± 130.37

Na	139.50 ± 1.18	142.40 ± 6.80	140.40 ± 0.70	141.20 ± 0.79

K	4.48 ± 0.98	4.36 ± 1.01	3.95 ± 0.78	3.99 ± 0.44

Cl	100.80 ± 1.40	102.30 ± 3.95	101.70 ± 0.67	101.40 ± 1.35

*Note*. ALB(g/dL), Albumin; ALP(IU/L), Alkaline phosphatase; CA(mg/dL), Calcium; CHO(mg/dL), Total cholesterol; CRE(mg/dL), Creatinine; GGT(IU/L), Gamma glutamyl transpeptidase; GLU(mg/dL), Glucose; AST(IU/L), Aspartate aminotransferase; ALT(IU/L), Alanine aminotransferase; LDH(IU/L), Lactate dehydrogenase; MG(mg/dL), Magnesium; TP(g/dL), Total protein; UA(mg/dL), Uric acid; BUN(mg/dL), Blood urea nitrogen; T-BIL(mg/dL), Total bilirubin; IP(mg/dL), Inorganic phosphorus; TG(mg/dL), Triglyceride; CPK(U/L), Creatine phosphokinase; Na(mmol/L), Sodium; K(mmol/L), Potassium; Cl(mmol/L), Chloride.

**Significant difference vs. control, *p *< 0.01

*Significant difference vs. control, *p *< 0.05

**Table 4:** Serum values for female rats after 90-day oral administration of silver nanoparticles (mean ± S.D.)

	Dose (mg/Kg)			
	
	0 (n = 10)	30 (n = 9)	125 (n = 10)	500 (n = 10)
ALB	2.78 ± 0.08	2.73 ± 0.10	2.75 ± 0.24	2.63 ± 0.09

ALP	237.50 ± 29.05	241.22 ± 22.12	253.50 ± 25.42	314.80 ± 42.13**

CA	10.42 ± 0.60	10.11 ± 0.26	10.08 ± 0.30	9.98 ± 0.27

CHO	107.50 ± 7.35	115.11 ± 12.36	116.30 ± 6.95	126.70 ± 12.39**

CRE	0.85 ± 0.16	0.96 ± 0.15	0.91 ± 0.10	0.90 ± 0.13

GGT	0.80 ± 0.42	1.11 ± 0.78	0.90 ± 0.57	0.80 ± 0.63

GLU	148.70 ± 20.09	146.33 ± 14.53	151.20 ± 16.48	151.30 ± 20.19

AST	94.80 ± 11.98	84.22 ± 23.82	84.20 ± 6.89	94.80 ± 38.14

ALT	67.70 ± 6.00	59.78 ± 10.57	61.10 ± 5.15	70.90 ± 22.00

LDH	722.00 ± 480.71	705.33 ± 612.27	496.90 ± 303.14	516.90 ± 245.26

MG	2.44 ± 0.17	2.30 ± 0.24	2.17 ± 0.08**	2.18 ± 0.15**

TP	6.30 ± 0.17	6.14 ± 0.22	6.08 ± 0.14*	6.03 ± 0.23*

UA	1.12 ± 0.39	0.97 ± 0.31	0.86 ± 0.19	0.87 ± 0.12

BUN	23.61 ± 2.57	22.26 ± 2.52	21.71 ± 1.94	23.03 ± 1.64

T-BIL	0.014 ± 0.008	0.022 ± 0.018	0.020 ± 0.018	0.019 ± 0.014

IP	6.50 ± 1.17	6.19 ± 0.99	5.43 ± 0.73*	5.32 ± 0.60*

TG	48.30 ± 24.07	55.22 ± 30.67	45.80 ± 19.99	50.30 ± 24.38

CPK	375.20 ± 290.40	1004.67 ± 2174.69	231.50 ± 124.83	245.70 ± 106.73

Na	140.10 ± 1.45	140.22 ± 0.67	141.40 ± 5.21	141.00 ± 1.05

K	4.02 ± 1.15	3.62 ± 0.73	3.40 ± 0.17	3.37 ± 0.26

Cl	102.20 ± 0.79	102.44 ± 1.13	103.60 ± 3.13	103.20 ± 1.69

*Note*. ALB(g/dL), Albumin; ALP(IU/L), Alkaline phosphatase; CA(mg/dL), Calcium; CHO(mg/dL), Total cholesterol; CRE(mg/dL), Creatinine; GGT(IU/L), Gamma glutamyl transpeptidase; GLU(mg/dL), Glucose; AST(IU/L), Aspartate aminotransferase; ALT(IU/L), Alanine aminotransferase; LDH(IU/L), Lactate dehydrogenase; MG(mg/dL), Magnesium; TP(g/dL), Total protein; UA(mg/dL), Uric acid; BUN(mg/dL), Blood urea nitrogen; T-BIL(mg/dL), Total bilirubin; IP(mg/dL), Inorganic phosphorus; TG(mg/dL), Triglyceride; CPK(U/L), Creatine phosphokinase; Na(mmol/L), Sodium; K(mmol/L), Potassium; Cl(mmol/L), Chloride.

*Significant difference vs. control, *p *< 0.05

**Significant difference vs. control, *p *< 0.01

**Table 5:** Hematological values for male rats after 90-day oral administration of silver nanoparticles (mean ± SD)

	Dose (mg/Kg)			
	
	0 (n = 9)	30 (n = 9)	125 (n = 10)	500 (n = 10)
WBC	8.33 ± 1.25	8.22 ± 1.44	8.17 ± 1.28	8.21 ± 1.07

RBC	8.98 ± 0.32	9.13 ± 0.66	8.88 ± 0.24	9.07 ± 0.35

Hb	16.67 ± 0.40	16.76 ± 1.10	16.47 ± 0.53	16.93 ± 0.68

HCT	36.96 ± 1.41	37.80 ± 2.90	36.97 ± 1.46	37.84 ± 2.12

MCV	41.17 ± 0.58	41.38 ± 0.72	41.62 ± 0.77	41.72 ± 0.92

MCH	18.58 ± 0.34	18.34 ± 0.40	18.55 ± 0.58	18.67 ± 0.37

MCHC	45.13 ± 1.08	44.38 ± 1.31	44.59 ± 1.69	44.79 ± 1.25

RDW	17.98 ± 0.78	17.83 ± 0.68	17.83 ± 1.04	18.10 ± 0.86

PLT	738.22 ± 53.33	731.56 ± 105.79	725.40 ± 45.43	718.50 ± 72.05

MPV	6.86 ± 0.29	6.84 ± 0.19	6.83 ± 0.27	6.64 ± 0.28

NEU	26.13 ± 3.30	26.72 ± 3.14	26.76 ± 4.14	27.12 ± 2.62

LYO	69.77 ± 3.39	68.21 ± 3.45	68.93 ± 5.30	69.34 ± 3.13

MONO	3.78 ± 0.80	4.57 ± 1.12	3.98 ± 1.19	3.35 ± 0.80

EOS	0.25 ± 0.40	0.39 ± 0.50	0.25 ± 0.21	0.15 ± 0.08

BASO	0.08 ± 0.14	0.12 ± 0.22	0.09 ± 0.08	0.04 ± 0.03

*Note*. WBC(K/μL), White blood cells; RBC(M/μL), Red blood cells; Hb(g/dL), Hemoglobin; HCT(%), Hematocrits; MCV(fl), Mean corpuscular volume; MCH(pg), Mean corpuscular hemoglobin; MCHC(g/dL), Mean corpuscular hemoglobin concentration; RDW(%), Red cell distribution width; PLT(K/μL), Platelets; MPV(fl), Mean platelet volume; NEU(%), Neutrophils; LYO(%), Lymphocytes; MONO(%), Monocytes; EOS(%), Eosinophils; BASO(%), Basophils.

**Table 6:** Hematological values for female rats after 90-day oral administration of silver nanoparticles (mean ± SD).

	Dose (mg/Kg)			
	
	0 (n = 8)	30 (n = 10)	125 (n = 10)	500 (n = 9)
WBC	5.46 ± 1.36	5.42 ± 0.81	5.11 ± 0.67	5.90 ± 0.97

RBC	8.46 ± 0.22	8.41 ± 0.34	8.19 ± 0.32	8.28 ± 0.24

Hb	16.24 ± 0.81	16.16 ± 0.75	15.82 ± 1.02	16.14 ± 0.85

HCT	34.45 ± 2.50	34.40 ± 1.80	34.33 ± 1.82	34.51 ± 2.13

MCV	40.70 ± 1.96	40.91 ± 1.62	41.95 ± 1.81	41.71 ± 2.45

MCH	19.20 ± 0.56	19.24 ± 0.81	19.34 ± 1.36	19.50 ± 0.96

MCHC	47.21 ± 1.80	47.02 ± 1.63	46.09 ± 1.96	46.84 ± 2.41

RDW	19.33 ± 2.44	19.18 ± 2.62	18.85 ± 2.55	19.04 ± 2.46

PLT	765.75 ± 110.30	718.30 ± 68.56	712.70 ± 55.93	688.89 ± 34.71

MPV	6.56 ± 0.71	6.74 ± 0.21	6.95 ± 0.28	6.70 ± 0.22

NEU	24.93 ± 2.88	27.37 ± 3.70	26.32 ± 6.70	24.06 ± 3.49

LYO	71.48 ± 3.83	68.32 ± 4.60	69.59 ± 7.53	71.22 ± 3.83

MONO	3.00 ± 1.17	3.85 ± 1.13	3.65 ± 0.79	4.54 ± 0.60*

EOS	0.42 ± 0.52	0.36 ± 0.17	0.39 ± 0.37	0.18 ± 0.15

BASO	0.17 ± 0.25	0.11 ± 0.09	0.15 ± 0.19	0.01 ± 0.02

*Note*. WBC(K/μL), White blood cells; RBC(M/μL), Red blood cells; Hb(g/dL), Hemoglobin; HCT(%), Hematocrits; MCV(fl), Mean corpuscular volume; MCH(pg), Mean corpuscular hemoglobin; MCHC(g/dL), Mean corpuscular hemoglobin concentration; RDW(%), Red cell distribution width; PLT(K/μL), Platelets; MPV(fl), Mean platelet volume; NEU(%), Neutrophils; LYO(%), Lymphocytes; MONO(%), Monocytes; EOS(%), Eosinophils; BASO(%), Basophils. *Significant difference vs. control, *p *< 0.05

**Table 7:** Reticulocyte counts for male and female rats after 90-day oral administration of silver nanoparticles (n = 5, EA/1000, mean ± SD)

	Dose (mg/Kg)			
	
	0	30	125	500
Male	10.00 ± 4.69	8.40 ± 4.72	6.10 ± 2.13	5.80 ± 2.74
				
Female	12.22 ± 4.84	6.70 ± 3.74*	12.10 ± 5.82	11.90 ± 3.90

*Significant difference vs. control, *p *< 0.05

### Histopathologic Examination

**Figure 4:**
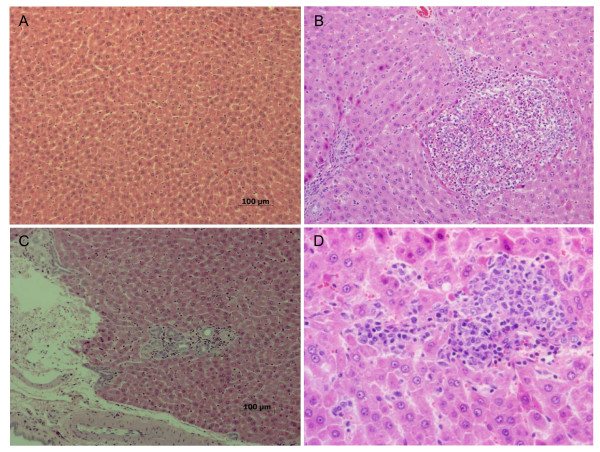
**Histopathological findings in liver: (A) control liver, (B) multiple foci of inflammatory cell infiltrates, including eosinophils, especially around central vein and portal areas (C) bile duct hyperplasia, and (D) several foci of inflammatory cell infiltration, especially around central vein and sinusoid on hepatic lobules**. Bar indicates 100 μm. A. Control (×100). B. High dose (×100). C. High dose (×100). D. High dose (×200).

In the liver, minimal bile-duct hyperplasia was identified in 4/10, 7/10, 8/10, and 6/10 of control, low, middle, and high-dose male rats, respectively (Table 8, Figure 4C). The higher incidence of bile-duct hyperplasia in treated male rats suggests a minimal effect due to silver nanoparticles in the treated groups. Minimal bile-duct hyperplasia was also present in 3/10, 7/10, 8/10, and 7/10 of the control, low, middle, and high-dose female rats, respectively (Table 9). Focal, multifocal, or lobular necrosis was noted in 0/10, 4/10, 5/10, and 4/10 of the control, low, middle, and high-dose male rats, respectively, and 0/10, 2/10, 2/10, and 2/10 of the control, low, middle, and high-dose female rats, respectively (Table 8 and 9, Figure 4B and 4D). The higher incidence of bile-duct hyperplasia, with or without necrosis, fibrosis, and/or pigmentation, in the treated animals also suggests a treatment-related effect. Although there appeared to be a slight increase of minimal tubular basophilia in the kidneys of high-dose male rats, these changes were not statistically significant and thus not considered to be test article exposure related. Tubular basophilia were also more prevalent in the male rats compared to the female rats (Table 8). Minimum or mild renal unilateral or bilateral mineralization was observed in 5/10, 8/10, 7/10, and 9/10 of the control, low, middle, and high-dose female rats, respectively, indicating a treatment-related effect.

**Table 8:** Histopathological findings for male rats after 90-day oral administration of silver nanoparticles

GROUP				Control		Low		Middle		High	
**Number of animals**				**10**		**10**		**10**		**10**	
				
				**N**	**%**	**N**	**%**	**N**	**%**	**N**	**%**

Liver	No microscopic findings			5/10	50	3/10	30	0/10	0	3/10	30
	
	Abnormality			5/10	50	7/10	70	10/10	100	7/10	70
	
	Hyperplasia	bile duct	minimum	4/10	40	7/10	70	8/10	80	6/10	60
			
			mild	0/10	0	0/10	0	1/10	10	0/10	0
	
	Vacuolation	hepatocellular	minimum	1/10	10	0/10	0	0/10	0	0/10	0
	
	Necrosis	focal	minimum	0/10	0	4/10	40	3/10	30	4/10	40
		
		lobular	moderate	0/10	0	0/10	0	1/10	10	0/10	0
		
		multifocal	moderate	0/10	0	0/10	0	1/10	10	0/10	0
	
	Hemorrhage			0/10	0	0/10	0	1/10	10	0/10	0
	
	Pigmentation			0/10	0	1/10	10	0/10	0	2/10	20

Kidneys	No microscopic findings			10/10	100	10/10	100	9/10	90	6/10	60
	
	Abnormality			0/10	0	0/10	0	1/10	10	4/10	40
	
	Basophilia	tubular	minimum	0/10	0	0/10	0	1/10	10	2/10	20
	
	Inflammation	focal	minimum	0/10	0	0/10	0	0/10	0	1/10	10
		
		tubular	minimum	0/10	0	0/10	0	0/10	0	1/10	10

Lungs	No microscopic findings			9/10	90	10/10	100	9/10	90	7/10	70
	
	Abnormality			1/10	10	0/10	0	1/10	10	3/10	30
	
	Inflammation	focal	minimum	1/10	10	0/10	0	0/10	0	1/10	10
	
	Histocytosis			0/10	0	0/10	0	1/10	10	0/10	0
	
	Mineralization		minimum	0/10	0	0/10	0	0/10	0	2/10	20
	
	Anthracosis			0/10	0	0/10	0	0/10	0	1/10	10

Intestines	No microscopic findings			10/10	100	10/10	100	2/10	20	2/10	20
	
	Abnormality			0/10	0	0/10	0	8/10	80	8/10	80
	
	Pigment	villi	yellow	0/10	0	0/10	0	1/10	10	8/10	80
			
			faint yellow	0/10	0	0/10	0	7/10	70	0/10	0

Heart	No microscopic findings			10/10	100	10/10	100	8/10	80	8/10	80
	
	Abnormality			0/10	0	0/10	0	2/10	20	2/10	20
	
	Inflammation	left ventricle	minimum	0/10	0	0/10	0	2/10	20	1/10	10
		
		right ventricle	minimum	0/10	0	0/10	0	0/10	0	1/10	10

Eyes	No microscopic findings			10/10	100	10/10	100	10/10	100	9/10	90
	
	Abnormality			0/10	0	0/10	0	0/10	0	1/10	10
	
	Inflammation	Harderian gland	minimum	0/10	0	0/10	0	0/10	0	1/10	10

Pancreas	No microscopic findings			10/10	100	10/10	100	10/10	100	8/10	80
	
	Abnormality			0/10	0	0/10	0	0/10	0	2/10	20
	
	Inflammation	pancreas	minimum	0/10	0	0/10	0	0/10	0	2/10	20

**Table 9:** Histopathological findings for female rats after 90-day oral administration of silver nanoparticles

GROUP				Control		Low		Middle		High	
**Number of animals**				**10**		**10**		**10**		**10**	
				
				**N**	**%**	**N**	**%**	**N**	**%**	**N**	**%**

Liver	No microscopic findings			5/10	50	2/10	20	2/10	20	3/10	30
	
	Abnormality			5/10	50	8/10	80	8/10	80	7/10	70
	
	Hyperplasia	bile duct	minimum	3/10	30	7/10	70	8/10	80	7/10	70
	
	Necrosis	focal	minimum	0/10	0	2/10	20	2/10	20	1/10	10
		
		central vein		0/10	0	0/10	0	0/10	0	1/10	10
	
	Fibrosis		minimum	2/10	20	1/10	10	2/10	20	1/10	10
	
	Pigmentation			0/10	0	0/10	0	4/10	40	1/10	10

Kidneys	No microscopic findings			5/10	50	2/10	20	3/10	30	1/10	10
	
	Abnormality			5/10	50	8/10	80	7/10	70	9/10	90
	
	Mineralization	unilateral	minimum	4/10	40	7/10	70	5/10	50	5/10	50
			
			mild	0/10	0	0/10	0	1/10	10	0/10	0
		
		bilateral	minimum	1/10	10	1/10	10	1/10	10	4/10	40

Lungs	No microscopic findings			10/10	100	10/10	100	9/10	90	10/10	100
	
	Abnormality			0/10	0	0/10	0	1/10	10	0/10	0
	
	Histocytosis			0/10	0	0/10	0	1/10	10	0/10	0

Intestines	No microscopic findings			10/10	100	10/10	100	10/10	100	5/10	50
	
	Abnormality			0/10	0	0/10	0	0/10	0	5/10	50
	
	Pigment	villi	yellow	0/10	0	0/10	0	0/10	0	0/10	0
			
			faint yellow	0/10	0	0/10	0	0/10	0	5/10	50

Eyes	No microscopic findings			8/10	80	9/10	90	9/10	90	9/10	90
	
	Abnormality			2/10	20	1/10	10	1/10	10	1/10	10
	
	Inflammation	Harderian gland	minimum	2/10	20	1/10	10	1/10	10	1/10	0

Pancreas	No microscopic findings			10/10	100	10/10	100	10/10	100	9/10	90
	
	Abnormality			0/10	0	0/10	0	0/10	0	1/10	10
	
	Inflammation	pancreas	minimum	0/10	0	0/10	0	0/10	0	1/10	10

Histopathologic examination of lung tissue did not show any treatment-related effects.

**Figure 5:**
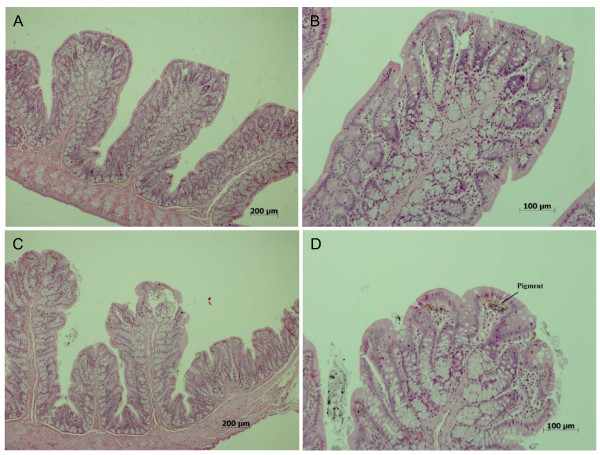
**Histopathological findings in intestines of male rats dosed with 500 mg/kg silver nanoparticles for 90 days**. A. Control male (×40). B. Control male (×100). C. High-dose male (×40). D. High-dose male (×100).

In the intestines, pigmentation of villi was observed in 0/10, 0/10, 8/10, and 8/10 of the control, low, middle, and high-dose male rats, respectively (Table 8). This dose-dependent increase in the pigmentation of the villi indicated an apparent treatment-related effect (Figure 5). In contrast, there was a slight treatment-related increase in pigmentation of intestinal villi in female rats (Table 9).

### Silver Distribution in Tissues

There was a statistically significant (P < 0.01) dose-dependent increase in the silver concentration of all the tissue samples from the groups exposed to silver nanoparticles in this study (Table 10). In addition, a two-fold higher accumulation of silver in the kidneys of female rats when compared with the male rats occurred across all the dose groups indicating a marked gender-dependent distribution.

**Table 10:** Tissue silver content after 90-day oral administration of silver nanoparticles

Silver (μg/g wet weight)						
	
Dose/Sex	Testes	Liver	Kidneys	Brain	Lungs	Blood
0 mg/Kg						
Male	0.04 ± 0.02	0.02 ± 0.01	0.04 ± 0.02	0.02 ± 0.01 ^a^	0.10 ± 0.08	0.001 ± 0.000
Female		0.01 ± 0.01	0.03 ± 0.01	0.01 ± 0.01 ^a^	0.05 ± 0.02	0.002 ± 0.002
						
30 mg/Kg						
Male	6.56 ± 0.33**	4.20 ± 1.57** ^a^	1.49 ± 0.37** ^b^	0.47 ± 0.18**	1.94 ± 0.64** ^b^	0.111 ± 0.016**
Female		8.56 ± 3.22** ^a^	7.98 ± 0.91** ^b^	0.38 ± 0.05**	4.97 ± 0.90 ^b^	0.087 ± 0.017**
						
125 mg/Kg						
Male	11.84 ± 1.62**	10.19 ± 2.09** ^b^	8.82 ± 2.13** ^b^	0.69 ± 0.06**	10.97 ± 3.81**	0.191 ± 0.037** ^b^
Female		29.13 ± 9.74** ^b^	37.09 ± 17.44** ^b^	0.77 ± 0.11**	17.64 ± 9.06**	0.122 ± 0.010** ^b^
						
500 mg/Kg						
Male	23.75 ± 9.13**	68.65 ± 33.59**	99.19 ± 32.82** ^b^	3.54 ± 1.44**	56.04 ± 51.14	0.419 ± 0.083**
Female		98.75 ± 31.65**	226.88 ± 55.64** ^b^	3.70 ± 1.17**	45.83 ± 11.43**	0.303 ± 0.099**

**Significant difference vs. control, *p *< 0.01. (ANOVA)

^a ^Significant difference vs. distinction of sex, *p *< 0.05. (T-test)

^b ^Significant difference vs. distinction of sex, *p *< 0.01. (T-test)

## Discussion

Silver nanoparticles are widely used as bactericidal agents in consumer products, but their potential effects in humans remain poorly understood. Silver at doses below levels that cause argyria or argyriosis are generally considered to be relatively non-toxic [12]. Wijnhoven et al. have hypothesized that the toxic effects of silver are proportional to free silver ions, but it is unclear how this relates to silver nanoparticles. Absorption of silver after oral administration has been shown to be subject to a first-pass effect through the liver, resulting in excretion into the bile [13]. Uncleared silver has been shown to be deposited in the renal glomerular basement membrane [14-17], mesangium [18], Kupffer cells, and sinusoid endothelium cells in the liver [15].

Unlike the 28-day study of Kim et al. [9], the present study shows intestinal pigmentation and effects from exposure to silver nanoparticles. Dose-dependent increases in the silver concentrations in the intestinal villi, as observed in this and other reports [19], and in the blood, indicate that the orally absorbed silver from nanoparticles is able to enter the blood circulation and be distributed to other organs. Since similar effects have not been previously reported for soluble silver, there is a possibility that these effects are due to particles as opposed to ionized silver.

Increases in renal silver reported in the current study are consistent with literature showing that ionized silver is deposited in the renal glomerular basement membrane [14-17] and mesangium [18]. The gender-related distribution of silver nanoparticles in the kidneys was also consistent with the results from the 28-day [9], 90-day oral, and 90-day inhalation studies [8]. The average nanoparticle sizes used in the 28-day and 90-day inhalation experiments were 15 nm and 18-19 nm, respectively, while the average nanoparticle size used in the 28-day and 90-day oral toxicity studies was 60 nm and 56 nm, respectively. Common treatment-related endpoints and distribution were found in all these studies indicating that distribution and toxicity do not appear to be dependent on particle size in the tested range or route of administration.

In previous reports, the target organs for silver nanoparticles were shown to be the liver in a 28-day oral toxicity study [9] and the liver and lungs in a 90-day inhalation study [8]. The results of the current study are consistent with previous reports with respect to the liver as the target organ and the tissue distribution of silver originating from nanoparticles. Liver toxicity as evaluated by histopathology included bile-duct hyperplasia and increased foci, which was consistent with the pathologic observations in the 28-day [9] oral toxicity and 90-day inhalation [8] toxicity studies. Increases in alkaline phosphatase and cholesterol were also consistent with the liver toxicity reported by Kim et al. and Sung et al.

In contrast to the 90-day inhalation study, no coagulation effects on peripheral blood were observed. Since there is similar hepatic pathology in the two studies, it is not clear whether increased coagulation times were due to hepatic injury in the inhalation study or some other mechanism.

Inhaled silver nanoparticles have been shown to cause lung inflammation [8]. These effects may be due to particle size since gold nanoparticles (which are not likely to be ionized) show similar effects [20]. Our very limited data makes it tempting to hypothesize that effects from silver nanoparticles are not only due to ionization of silver from the surface of silver nanoparticles, but may originate (at least in part) from direct effects of nanoparticles. The relationship between particle effect and effect from ionized silver could be tested by quantitatively examining target tissues for the presence of nanoparticles with a concurrent measurement of tissue ionized silver as opposed to measuring total tissue silver alone. If silver nanoparticles have particle-related effects which are tissue specific and significant compared to effects from ionized silver, it will be important to incorporate such differences into future occupational and environmental risk assessments.

## Conclusions

A LOAEL (lowest observable adverse effect level) of 125 mg/kg and a NOAEL (no observable adverse effect level) of 30 mg/kg are suggested from the current 90-day oral toxicity study. The LOAEL from this study is significantly lower than the LOAEL of 300 mg/kg derived from the 28-day oral toxicity study (Kim et al., 2008). However, the NOAEL derived from this study is consistent with the NOAEL of 30 mg/kg derived from the 28-day oral study (Kim et al., 2008).

## Competing interests

The authors declare that they have no competing interests.

## Authors' contributions

IJY headed the study and performed pathologic analyses together with MYS, YHC, HKC, IHK and drafted the manuscript with YSK and BK. YSK headed all animal treatments with MYS, KSS, and HRR. JDP and JHL contributed to the distribution study. IJY, BK and KHO conceived and designed the study. All authors reviewed and interpreted data and read and approved the final manuscript.
